# A Résumé of Fifty Years’ Obstetric Practice1Read before the Buffalo Academy of Medicine, January 26. 1897.

**Published:** 1897-06

**Authors:** John Hauenstein

**Affiliations:** Buffalo, N. Y. 309 Elmwood Avenue


### A RÉSUMÉ OF FIFTY YEARS' OBSTETRIC PRACTICE.[1]

By JOHN HAUENSTEIN, M. D., Buffalo, N. Y.

A FRIEND of mine who commands a prominent position as an obstetrician in this city, suggested that it were very desirable if the older members of the profession should volunteer to relate some of their experiences to their colleagues, which might result in benefit to some, particularly to the younger and less experienced, who are engaged in the practice of obstetrics.

By reason of my friend's suggestion, and the fact of having had rare opportunities of acquiring some experience in the practice of midwifery, since I was called by almost every midwife on the east side of this city during a period of at least twenty years, to assist them whenever they had a case of difficult labor on hand which they could not well manage themselves, besides by quite a number of physicians who required assistance, I have concluded to give a brief sketch of my experience on a few subjects relating to the management of the parturient.

*Anesthetics.*—When I commenced the practice of medicine, and for several years after, anesthetics had not made their advent and patients had to suffer the brunt of all painful operations, however tormenting. Some luxations were the dread of the surgeon. Now, under the influence of an anesthetic, no complaint is made by the patient, and moreover, at the same time, some relaxa-

1. Read before the Buffalo Academy of Medicine, January 26, 1897.

tion of the tissues is achieved favorable to a reduction. In obstetric practice, however tormenting the suffering and the pain of labor, or during an operation necessary to correct some malposition of the child, great as the suffering of the parturient might be, we had no means at hand to soothe the pain effectually.

To contemplate the change which has taken place and the boon which has been contributed by anesthetics to ameliorate the suffering of the unfortunate, is simply phenomenal. No one can appreciate the blessings thus conferred more than he who had the opportunity of witnessing the torment of pain in the various distressed conditions, the dread and suffering in what were painful operations before the introduction of anesthetics, compared with the same conditions and the same kind of operations under their influence today, with no manifestations indicative of pain.

Ether was somewhat slow in asserting its claim as an anesthetic for the plausible reason, I believe, that Dr. Morton, when he realised the great importance of his discovery, was captivated and bewildered by the prospect of a dazzling pecuniary reward; then he made the mistake of procuring a patent, knowing, as he must have known, that patents were not favored by the medical profession. This mistake reverted to his disappointment in the realisation of his hope and made him an unhappy man. Thus it happened that his discovery did not meet with the enthusiasm which otherwise would have been in store for him, and that chloroform, which made its advent a year later, spread like wildfire in its adoption as an anesthetic, because not encumbered by a patent. Chloroform maintained its reputation in this city and elsewhere up to a very recent time, until assailed and made the scapegoat of many sins. But I have reason to believe, from observation and practical experience, that carelessness and a want of the necessary precautions were frequently as much to blame as the chloroform itself in causing the mishaps. I have administered chloroform very many times in my obstetric practice, as well as when requested by surgeons to administer it, which happened frequently, but I never observed any serious mishaps.

However, chloroform has yet claims to be employed in most obstetric cases in preference to ether, and its use in such emergencies will be continued for a long time yet to come.

My friend, Dr. Van Peyma, tells me that so far no case of death from chloroform, in the practice of obstetrics, is reported. This observation, together with the fact of the apparent suffering, frequently observed and manifested by moaning, writhing and contortions, in the first stage of the administration of ether, should incline, almost exclusively, to the use of chloroform in obstetric cases.

The two most important precautions against accidents from the use of chloroform, omitting a number of others of less moment, are slow and gradual administration and with plenty of air, which can best be accomplished, in my opinion, by the use, for the purpose, of a simple napkin folded and held off a little from the mouth and nose so as to admit air, thus avoiding the cone which prevents the admission of the requisite quantity of air. The waste of chloroform by this means is of no consideration when compared with safety. The mask, which I have not used, is also considered by some as a safe medium for its administration, admitting, as is claimed, a sufficient quantity of air.

*Antiseptics.*—The triumph among the numerous achievements in medicine of the past half century occurred when antiseptics came to join hands with anesthetics and formed a partnership, the result of which revolutionised the domain of the practice of medicine and particularly that of surgery. Septicemia, childbed fevers and all such diseases as are infectious, whose germs find a nidus and consequent entrance into the circulation, with strictly antiseptic precautions can now be almost entirely prevented. This is preventive medicine and together with what bacteriology has in store, the future promises a brilliant field for the practitioner of medicine and surgery which will supply him with a rich harvest of resources that will enable him to prevent disease.

To be provided with the weapon by which he is enabled to stay such a fiend as childbed fever, the physician can well be proud of his profession. I have witnessed two epidemics (?) of this disease, which is highly infectious and dangerous, and many were the victims that were infected by the midwives, the patients of some of whom almost invariably took sick with the disease. I remonstrated and even threatened with prosecution at law if they would not desist from attending obstetric cases. But at that time they were not under control and, with few honorable exceptions, were an ignorant group. They were then not compelled to pass an examination as now, and the health department was inefficient to enforce sanitary measures. Today a note of a few words of complaint to the health commissioner would make harmless these criminals.

Effective, as a rule, as is the antiseptic treatment of preventing infection of the patient, it behooves the accoucheur to be on his guard so as not to become himself infected with a loathsome disease ; for such is my sad experience that I can bear evidence of the danger of infection to the obstetrician under certain favoring circumstances and conditions. I was called by a physician to assist him in a case of delivery. After inquiry, but before an examination, I disinfected my hands with a solution of bichloride of mercury, then on introducing my finger into the vagina, I found the woman in labor with a fetus which proved afterwards to be of about the fifth month, in a decomposed and putrescent state, the lower extremities presenting, with not quite enough dilatation of the os uteri, to permit its passage. With my index finger and thumb I took hold of the feet of the fetus and made gentle traction for quite a while, when it was born. In due course of time I had two typical chancres, one on my thumb the other on my index finger, and to make the diagnosis doubly sure I subsequently had the secondary syphilitic eruption. About the same time the woman called at my office and showed me her crop of eruptions. This brief notice of my unpleasant experience of the dangers to the accoucheur of becoming infected with a loathsome disease deserves reflection.

*The Knee-Chest Posture*, which has for its object the reversion of gravitation as regards the uterus and its contents.—I find some difficulty in fixing the exact time when first the knee-chest posture was introduced and practised in this country. Robert Barnes, London, 1876, on the subject of prolapse of the cord says : "The resort to the knee-elbow position is not altogether new. I was taught it by the late Dr. Bloxam, nearly thirty years ago, but it has lately been enforced, and successfully applied by Dr. Thomas, of New York, under the name of the 'postural method,' (Transaction of the N. Y. Academy of Medicine, 1858); W. Theopold, (*Deutsche Klinic*, 1860) ; by Dr. Wilson, (*Glasgow Medical Journal*, 1867,) and others." From these statements must be concluded that the postural position was not known and much less applied by the obstetrician in this as well as in countries in Europe before the year 1858, except by a very few, if we accept the remarks of Dr. Barnes.

The knee-chest posture is of vast importance and advantage in the management of a number of cases of difficult labor. It becomes almost absolutely necessary in the successful management of prolapse of the cord, to reposit which great reliance can be based upon the assistance of gravitation. Equally advantageous is the knee-chest posture in face presentations to correct the position of the child's head by bringing up the vertex.

The new method which I have never employed, because I knew it not, which has for its object the same principle—namely, that of gravitation, is the method of Trendelenberg, which prescribes a posture in which chloroform can be administered, an advantage in some cases, since the knee-chest posture is not well adapted for its administration.

*Prolapse of Cord.*—This accident receives its great importance from the published statistics, according to which more lives have been sacrificed formerly, for the want of knowledge of its management as now established in this country as well as in Europe, than any other accident incident to the child of the parturient. Considering that about 2,000,000 of births take place annually in the United States and that, according to reports, in 250 or 260 births one of prolapse of cord happens, and that nearly one-half the children of this number are born dead, one can contemplate the great significance of this subject. This startling percentage of deaths can and ought to be reduced to a minimum, according to my experience, and in consideration of the facilities at the command of the obstetrician today, by means of which the cord can be returned readily in the knee-chest posture by the hand, beyond the head of the child. The knee-chest posture offers two very great advantages, in that it permits the hand easily to pass by the head of the child and that by the law of gravitation the cord sinks almost of itself to the fundus of the uterus in this position.

As regards the mode of procedure to be followed in returning the prolapsed cord, the opinion prevails, almost unanimously among the authorities, that the hand should be used. The *American Text-Book of Obstetrics*, 1895, says: "There is no instrument equal to the hand for this purpose" and discards entirely the use of instruments. I could quote from more authorities, but I deem it not necessary to enforce the proper procedure. There is one writer, Ed. Reynolds, M. D., however, (*Practical Midwifery*, 1892,) who furnishes unction to those that still continue to dilly-dally and fumble with the catheter, or some one of the scores of similar instruments, and a ribbon, with which they may sometimes succeed in returning a prolapsed cord. He says: "In most cases, however, digital reposition fails, and in such cases an attempt should be made to replace the prolapsed portion of the funis by instrumental means." This opinion is undoubtedly based upon a want of practical experience, for it is contrary to the views of almost all the authority on this subject, and decidedly contrary to experience, and although I claim no authority, yet I presume to deny the correctness of the view.

I can think of but one case in which an instrument could assert a claim, and that is in a case of insufficiency of dilatation of the os uteri to admit the hand, and yet attended with danger of compression of the cord, but the introduction of the instrument with the portion of cord prolapsed ought to be made in the knee-chest posture, as absolutely necessary. I have never met with such a case.

I firmly believe that in the near future the obstetrician will have acquired such dexterity in the manipulation of these cases that the statistics will show a minimum in death-rate of children. My opinion finds support in that some obstetricians, by the improved technique, have already materially reduced the death-rate.

Ever since the knee-chest posture was made known in this country, I practised the method of returning the cord as now generally applied. I have treated quite a number of cases, more than is generally allotted to one physician during his practice. In most instances I was called by midwives and found, almost invariably, strange as it may seem, the cord beating and the os uteri sufficiently dilated for the hand to pass.

The manner of my procedure was, whether it were simply a loop or a handful, to take the portion of prolapsed cord in my hand, right or left, as the case required, the patient being in the knee-chest position, and pass it down beyond the head, keeping the cord with the end of my fingers in place until the patient was turned on her back, making some pressure, with the free hand, externally on the head of the child towards the pelvis and then withdrawing my hand from the uterus. I then placed my ear on the woman's abdomen and if I found the fetal heart-beat to be satisfactory my services were concluded. I never gave chloroform. However, if chloroform has to be given, the patient may have to be held in the postural position by assistants, or in the new postural position, that of Trendelenberg. Since I adopted this method I do not remember a case which did not prove successful in preserving the life of the child.

*Face Presentation.*—I have some remarks yet to make on this accident in addition to what I said in a paper which was written as long ago as 1881, and which was read in a meeting of the Medical Society of the County of Erie in January, 1882. I claim to have been one of the first, if not the first, to take advantage of the postural method in performing cephalic version in face presentations, and I am sustained in my assumption because the literature on this topic as late as at the time I wrote the paper mentioned was barren on this subject; for I could find nothing in the text-books and treatises on midwifery that portrayed any similarity to the method I had employed since the knee-chest posture became known in this country.

Although the object of correcting a face presentation is not so much for saving life as it is to alleviate pain and suffering and shorten the duration of labor, yet cases of death continue still to be reported from time to time as a result of the accident. In this connection I will mention a case that occurred in this city a number of years ago, which was related to me by the physician whose case it was, and who called as consultant and assistant to another, who at the time had one of the most extensive practices in midwifery in the city, but in spite of their efforts, strange as it seems, both mother and child were permitted to die.

When this physician, whose case it was, called me some years later for assistance in another case, in which I made flexion of the head in the knee-chest position, he was so delighted that he prevailed on me and insisted that I should publish the method, which I did as you know.

The literature of today, as I am told by a friend, a specialist on obstetrics, who is well-informed on the subject, contains over one hundred articles published in the medical journals, treating of face presentations, particularly as regards the cause and the *modus operandi* of its occurrence. All such speculations and theories as to the cause of face presentations possess no value in a practical point of view. It is immaterial by what mysterious passage or freak of nature the presentation might have been effected, unless the means at the same time can be pointed out by which the occurrence can be prevented, for when the accoucheur meets with a face presentation it is there, and no speculation or theory will alter it, but according to my judgment, based upon some experience, it becomes his duty to correct, if possible, the malposition of the head.

The superabundance of literature on this subject; the differences of views existing among writers as to the propriety of interference, unless in exceptional cases, some trusting to nature almost exclusively to accomplish labor, others considering it quite a serious occurrence that requires interference and correction if possible; the various methods of treatment and management recommended mystify the subject to a degree that the sarcastic proverb or saying of the Germans becomes expressive of the situation, which translated is: "On account of the multitude of trees one cannot see the forest." I have no doubt that many of these productions deserve to be relegated to the waste basket.

But, notwithstanding, that face presentations have been so much written about of late years and brought to the notice of the profession so generally and amply, I have found no notice of the postural method for the correction of the accident in any of the text-books, although I have examined a number of treatises of American, English and German authors, excepting in a lecture by Otto von Weiss, Vienna, published in Leipzig, in pamphlet form of 42 royal octavo pages, 1893, in which mention is made of Humphrey, *American Journal of Medical Science*, 1877, who brought down the vertex in the postural position, and Partridge, *American Journal of Obstetrics*, 1884, who succeeded four times in five with the version. Also Bayer, (*Sammlung Klinischer Vorträge*, Volkmann, No. 278, 1886,) twice in three face presentations ; in the third case with a rachitic deformed pelvis he was compelled immediately upon rectification to use the forceps to prevent the return of the deflection.

Many describe a proceeding by manipulation, both of external and internal, to bring down the vertex, but with only exceptional success. The forceps and podalic versions were frequently resorted to.

It is somewhat inexplicable to me that the treatment of the prolapsed cord in the postural position should be so generally adopted and the various, mostly unsuccessful, attempts to correct the position of the head in face presentations should still be continued, particularly since the object in view is achieved almost as easily in the one case as in the other.

The fog which seems to surround the subject caused by the various and discrepant views in the books of obstetrics ought not to deter any physician from the attempt and performance of cephalic version in the postural position in any case to which he may be called and in which interference is indicated ; and it is indicated in almost all cases, for even measurement and palpation are not always reliable and during the time such measurement requires, the version can be accomplished without much inconvenience or pain to the parturient. There is, however, one exception that may and ought to be taken into consideration and that is when the head is deep down and the chin anterior; such a case can be left to the efforts of nature or, if necessary, to the use of the forceps.

A few words more regarding the mode of operation, a repetition of what I have already said in my paper referred to. The patient being in the knee-chest position, if the head be already engaged in the pelvis, it can generally, readily be pushed down toward the fundus of the uterus, aided as it is by gravitation. The hand, right or left as may be required by the position of the face, can then easily be passed down beyond the head and the vertex brought up to the position of a vertex presentation. The only difficulty frequently encountered in the manipulation is the slipperiness of the scalp, which prevents a firm hold, but it can be overcome. I have been successful in accomplishing the version in all cases to which I was called, with the exception of one case of which Dr. Van Peyma reminded me which occurred many years ago; and what I can do others can do who are gifted with a little dexterity in manipulation.

After reviewing the subject calmly as regards the different methods of management, reported by quite a number of authorities, also in consideration of the many deaths reported in the pamphlet referred to, particularly in brow presentations, which by the postural method are treated like face presentations, and the advantage of the conditions of an easy labor achieved by the flexion of the head, I am of the firm opinion and belief that in the near future the postural method to rectify face presentations will generally be adopted as preëminently the method in the treatment of this accident.

*The Forceps.*—My first case of forceps delivery impressed itself so vividly upon my memory that I have frequently since been amused by the recollection of it. In those days when I attended lectures the opportunities which a hospital connected with a medical school presented for clinical instructions were few. I was a novice in the use of the instrument, never having seen it applied on the living subject or received any instructions except those on a manikin in the lecture room from my seat at a distance.

She was quite an intelligent midwife and one that understood her profession very well, a compliment which then but few deserved, that called me to my first case of forceps delivery. As you can imagine, I did not feel very comfortable, for to be a doctor it is expected that he should know a little more than a midwife and be in command of some little skill in the manipulation of the instrument. However, I began fumbling and groping about in perplexity for a long time, and if she had not had the patience of a Job she would have discharged me and sent for some one else. But at last I succeeded in delivering a live baby. Then I was elated and concluded to do better the next time.

The forceps is an absolutely indispensable instrument in the hands of an obstetrician to enable him successfully to perform his function. It is the *sine qua non* in his armamentarium of useful means of overcoming difficulties, which in many cases, without it, would determine a fatal result.

The scope of my paper, however, permits but a limited treatment of this subject, and I must therefore deny myself the privilege of recommending or pointing out the particular instrument, and the essential parts of a good forceps among the many models best adapted for the purpose of the accoucheur. Besides, it requires a familiarity with the different instruments on the market and in use, which I do not now possess. During the longer time of my practice I used the Hodge forceps, which answered my purpose very well. I used also the White forceps occasionally, and when the resistance was not too great it proved to be a very acceptable instrument, but in cases when considerable force was necessary to be used it was apt to spring, but I am now told that this defect has been remedied.

The axis-traction forceps I have used only a few times and found it a little more cumbersome in its application than the simple instrument, and as regards traction it offers no appreciable advantage to one who is conversant with the axis of the curve of the pelvis, but may assist the unpractised in that of showing him the axis of the curve. It seems to me, therefore, that in the case of the simple forceps the operator shows the instrument the direction in which traction should be made and in the other case the axis-traction forceps shows the operator the direction. I have succeeded in delivering a child alive with my simple forceps when the axis-traction forceps in other hands failed.

The forceps nowadays used are a decided improvement on those that were formerly employed, although the American forceps, fifty years ago, was fairly well adapted for this purpose compared with some European forceps which I have seen. I had intended to show you a forceps which has a dangerous appearance, yet there there is no doubt but that formerly, fifty years ago, it was used in some parts of Germany. It was given to me by the late Dr. Brunck, who in this city practised medicine in the early forties until he assumed the editorship of the *German Democrat*. I presented it to Dr. Mann ; it is entitled to a place in a collection of medical instrument curiosities and you may have an opportunity sometime to see it in such a collection.

The other example is in a foreign land and will never come to our shores except as a curiosity. When in Europe, in 1881, I visited ten or twelve days with a doctor in Alsace, a cousin of my wife. One day during my sojourn he asked me to accompany him to a case of labor in an adjoining village, not as a consultant, however, which I very cheerfully accepted. On arrival we found a woman in her first labor, which was progressing rather slowly, attended by a midwife. He was anxious to show me attention, and dinner-time drawing near, he concluded to expedite the labor by employing the forceps. He proceeded to apply the forceps and did it very well, making use of moderate traction, as I judged, and the child was brought in due time into the world, but dead. I tried for a long time to resuscitate it, but to no purpose. It seemed strange to me that with the ease and moderate amount of force apparently employed in its delivery, the child should be born dead. However, upon examining his forceps, the riddle, to my satisfaction, was solved. It was a heavy piece of iron, I judged it about 3½ pounds or more in weight, with no spring or elasticity of its blades—a veritable cranioclast. Anemia of the brain or extravasation of blood from pressure, I conclude, to have been the cause of its death.

It was a French forceps, for he was so thoroughly a Frenchman that he would use no other. The use of such an instrument but a few years ago, which is doubtless yet used in some parts of rural France, does not speak in very complimentary terms of the highly educated and intelligent medical profession of that country as regards improvements of modern appliances in the obstetric art. In the use of the forceps I have witnessed two extremes of traction or force employed when in the early days of my practice I had occasion to call for assistance. In the one case the assistant called would not cease with a continued and uninterrupted pull, until the head of the child was born ; the other would dilly-dally an entire night with a little traction now and then, whenever a little pain manifested itself. It is unnecessary to say that both procedures are faulty.

I read some time ago of a certain professor who said if not in my words, at least in sense, that you must never use force with the forceps, which reminds me of what I have to say about the use of force. The exercise of a little charity, however, will explain the meaning of the professor's advice, which he probably intended to convey. To interpret the advice as meaning inaction with the forceps applied and in hand, would be as absurd as to expect, in this situation, that the child would be born by whistling "Yankee Doodle came to town." What he doubtless meant to say was to use very moderate traction. But even with such charitable interpretation my experience does not agree. There are cases in which a great deal of force will have to be used, but when the forceps is properly adjusted and traction made in the right direction, I have never seen any harm done the woman.

But it is not the integrity of the parts of the woman alone that demands the exercise of judgment and prudence; there is a life imperiled and in danger by the forceps, and in order to save the child the facts call for the utmost precaution as regards traction and particularly as regards pressure. To prevent, if possible, fatal anemia of the brain or extravasation of blood, traction ought to be interrupted for a few moments, from time to time, so as to give the blood some chance to reënter the brain again.

But if a forceps delivery excludes the hope of saving the child's life (and yet, some survive almost a heroic operation with the forceps) and craniotomy is considered unjustifiable, then in order to save the life of the child the question confronts the accoucheur which of the two methods he shall adopt, the Cesarean operation or. symphyseotomy, both grave and hazardous, shorn in recent time of some of their danger by antiseptic precautions. Symphyseotomy is condemned by the German obstetricians generally, and I am inclined to believe that it will never be adopted to any considerable extent by the profession. But it is a feather in the hat of him who succeeds in saving a woman and child by it and this doubtless contributes the most to sustain its questionable claim.

In case of pendulous abdomen it becomes sometimes necessary to use the forceps. I have, however, succeeded in such condition to assist nature in accomplishing labor without further interference, by tying tightly a sheet around the pendulous abdomen and over the neck of the patient, thus assisting in bringing the head into the pelvis. But if the forceps must be resorted to, the application and adjustment is not so easily done as in ordinary cases where the vertex can be readily reached. In such a case of pendulous abdomen it is well to have the assistant hold the first blade, which is adjusted in a perpendicular direction until the second blade is adjusted, then if traction be made it must first be made downward and a little backward until the pubic arch is cleared or overcome. The forceps in such a case will then have described three-quarters of a circle, as can be seen if left adjusted until the head is fully born. A little practice in manipulation is required. Except where deformity of the pelvis exists these cases are not met with in the primipara.

In ordinary cases it is an easy performance to adjust the forceps, although my first case would not seem to justify this statement. However, cases of perplexity have happened and will doubtless yet happen, such as the case to which a physician, a member of the Medical Society of the County of Erie, and one possessed of considerable intelligence once called me for assistance. He had tried hard for some time to apply the forceps but did not succeed. Upon examining I found that he had pushed the blade against a fold of the os uteri and consequently failed in his efforts. This case teaches that in the application of the forceps one must keep close to the head of the child with the blade of the instrument introduced, then this difficulty will be avoided in its application. I have used the forceps many more than a hundred times, and in some cases I was compelled to use considerable force, but I remember no case in which I used it that I regret having done so. But I do remember with regret a case in which I did not use it, for I did not have it at hand, the case being six miles away from the city. The reason I did not have the instrument with me was that the professor of the school in which I attended lectures put particular stress on his injunction never to take the forceps along when called to a case of labor, and I, as a dutiful alumnus, left it at home. The patient was a fleshy woman and no longer young; labor proceeded fairly well and regularly, but not as expeditiously as I wished. After a time she began to have very strong pains, but with no advancement of the head. I thought of my forceps with regret that I did not have it with me. At last I sent for it, but alas! before it arrived the woman made an exclamation characteristic of rupture of the uterus, and so it was. I sincerely regret as a partner of the professor my part of the criminal neglect, for after all, the woman living so far away from the city, I ought to have ignored his instructions. This case happened in the very early days of my practice.

*Transverse Presentations.*—Among the large number of difficult labors to which I was called there were, of course, a proportionate number of transverse presentations, of which a few interested me particularly.

I was called by a midwife to a case of transverse presentation, and upon inspecting the abdomen of the parturient I was at once tempted to make version by external manipulation, the woman being much emaciated, by reason of which every part of the child was distinctly outlined and discernible to the eye. No palpation was necessary to determine its position, and the ease with which I succeeded in turning it surprised me and the midwife exclaimed that I must be a wizard. This one case convinced me that even in less favorable conditions the attempt to correct by this method the position of the fetus in utero ought to be made much oftener than formerly.

Another case to which a midwife called me for assistance terminated not so happily. When I approached the patient for an examination I observed an arm protruding through the vulva. On inquiring as to how long this condition had existed, she answered, "Quite a while. I pulled on the arm for some time, but I did not succeed in bringing down the child." I looked at the patient's face and its anxious expression indicated a grave condition of her case. On a closer examination I found her pulse beat 150 or more and very feeble, a despondent expression was depicted and altogether the appearance and symptoms indicated that something very serious had taken place. She was really in a collapse, and how to account for it except on the hypothesis that she had sustained a rupture of the uterus was not evident. In order to protect myself against the suspicion and inference that I might myself have been instrumental in having caused her sad condition, before I touched her for the purpose of relief I requested the attendance of another physician to be called at once to assist me. I waited some time, but no physician came. I then explained the condition of the patient to the husband and concluded, in defiance of the desperate situation and hopeless result that seemed inevitable, to turn and bring the child away, which was accomplished with little difficulty, then to finish labor and remove the placenta. When on introducing my hand into the uterus for the purpose it came in contact with the intestines of the patient. This was a plain case of malpractice and the midwife deserved punishment, but the husband, being a clergyman, protested and begged me not to report it.

In a third case I was called by a physician. I found that the trunk of the child had already been brought down, but the chin was hooked against the arch of the pubis ; the forceps had been applied, but all the pulling could not dislodge it. By pushing the chin up and turning it to one side the difficulty was overcome and the child was born. The physician was a beginner and therefore had not yet acquired much practice nor had any experience. Today, I am sure, such an oversight could not occur to him. It made me think of my first case of forceps delivery ; he doubtless also recalls the case to memory with a smile.

From the fact, already stated, of being so frequently called to cases of difficult labor, I have met with a number of transverse presentations which required turning as the only feasible alternative in such cases that permit a child being born, but I do not remember any marked or very unusual occurrences in addition to what I have already mentioned in any of the cases I so treated that deserve mention.

Turning is frequently easily performed. At other times and in some cases it is attended with some difficulty, depending mostly upon the length of time that has elapsed since the liquor amnii drained off, and the degree of tonic contraction of the uterus ; it may, therefore, happen in some cases that the operator earns his fee richly.

Permit me now the privilege of a short digression in relating as an episode to the previous subject an interesting case, not for the purpose of instruction and much less to be imitated, but as an example of heroic midwifery. Although I was not an eyewitness and had no hand in it, the facts are, nevertheless, reliable because they were stated under oath. Soon after I engaged in the practice of my profession, a self-styled doctor from Germany came and settled in this city for the purpose of practising medicine. He was rather an undesirable competitor, since he possessed the qualifications of a charlatan in a high degree and made use of them ; by word of mouth, in newspaper articles in which he would parade some remarkable cure he had made, by demeanor, and in fact by all the means at his command he would endeavor to force himself upon the attention of the people. To give you an instance : He was called down town near the docks to a laborer who had received an injury to his foot of not a very serious character, as I learned afterward, but, nevertheless, he bandaged it in a demonstrative way, had him laid on a truck with a man beside him holding up the injured foot high in the air, and in this position had him moved from the docks through Main street up to Genesee street, then home. The doctor, like Sancho Panza on his ass Dapple, following the knight of Don Quixote's fame, followed his patient high on horseback closely behind on the journey. However ludicrous the performance may have appeared to some, he nevertheless achieved his object, the talk of the town, since for a while his name was in everybody's mouth.

But the finale of the drama in which he played such a conspicuous rôle was near at hand, and as the popular saying goes, "each dog will have his day," was soon to be verified in his case. A countryman of the same people in whose family the act took place related the circumstances to me, which briefly told were: the doctor in the attempt to deliver the woman of the family, pulled the trunk off from the head of the child and that the head remained undelivered and was buried with the corpse of the woman and trunk of the child.

To suppress an opportunity so favorable for exposing a pretender could hardly be expected of me, considering the feeling and sentiment which I must have entertained against a rival who resorted to such unprofessional means as I have mentioned to acquire practice and a reputation. But regardless of whatever opinion on the subject may be entertained, frail human nature asserted its course and I reported the case to the coroner, who had been my preceptor. The victim was exhumed and a thorough examination made by a prominent physician, measurements of the head of the child and pelvis of the woman and so forth were taken, all preparatory to a legal prosecution.

On the trial I was summoned as interpreter, but it had leaked out that I was concerned in the instigation of the prosecution and the defendant's counsel objected to my acting as interpreter, but did not prevail, for the District Attorney, George P. Barker, a lawyer of high standing, insisted that I should be retained as interpreter and so the judge decided. The witnesses belonged to an ignorant class of immigrants, and spoke the Allemanian dialect only, which is not generally well understood by those who speak the German language proper. But as I had spoken that tongue in my boyhood I understood it quite well and this fact may seemingly have come to the ear of the court.

The trial lasted a number of days and excited a great deal of interest in the community, for the court room was crowded with spectators each day during the trial. The sentence of the court was that defendant be confined in the Erie County Jail for one year, according to the law then in force.

*The Placenta* is the source of much trouble and vexation to the obstetrician both near and at full term and in abortion cases. A large volume could be profitably written on the different phases of the accidents incident to it, but it does not come within the scope of this paper to say anything about the annoyances of the placenta in abortion cases, and as regards its features at near and full term, I shall touch upon only a few of the most prominent frequently met with in obstetric practice.

In the very early time of my practice I was called by a midwife to remove a retained placenta, the patient living about four miles from the city. On arriving at the bedside of the patient I introduced my hand into the uterus and in a few moments it was clinched by the tonic contraction of the uterus so powerfully that it became benumbed and I could not tell whether I had that hand or not. Chloroform had not yet made its advent and no assistance was to be had in the neighborhood, and the saints did not answer my call ; at the same time the woman was weakening from loss of blood. Under such circumstances I was in perfect mental agony and the time seemed an age before I could stir my hand to advantage. At last, after a long time, I succeeded in removing the placenta. Many years afterward I was called by a physician whom I found exhausted by the bedside of the patient from the efforts of removing an adherent placenta. The entire disk was firmly adherent and it required a long time to saw it off, as it were, with the edge of the hand and tips of the fingers.

*Placenta Previa* is doubtless one of the most unfortunate accidents to a woman with which the obstetrician can meet. In consequence of the many midwives that called for my assistance whenever they had a case of difficult labor which they could not well manage themselves, I had the unpleasant experience to meet with a larger number of placentæ previæ than is commonly allotted to the general practitioner.

The management of a case as now recommended which includes early turning, slow extraction, antiseptics, is the very reverse of that formerly practised. Then early turning was out of question, unless in exceptional cases in which the os uteri was sufficiently dilated to admit the hand, since Braxton Hicks's method was not yet practised, and I doubt whether it was then known. Turning was delayed until the os uteri admitted the whole hand, during which delay a dangerous loss of blood frequently occurred, imper iling the life of the woman. Delivery was accomplished immediately upon turning and the sudden emptying of the uterus in the enfeebled condition of the patient sometimes induced a shock that proved fatal. Antiseptics were not used for the obvious reason that they were not yet at hand.

But as the American Text-book of Obstetrics truly says: "There is no single method of treatment in placenta previa applicable in all cases and at all times." And I may add the treat ment rests in great part upon the good or bad judgment of the obstetrician in charge.

Nagel says "That in some years placenta presentation was so frequent that it seemed as if it were almost epidemic." The unhappy results were many, as the statistics show, and I had my share of them, I regret to say.

Among the authors that give a collection of cases and the percentage of deaths to mothers, the death-rate of children being higher, but which I have omitted: King, 22.5 per cent.; Müller, 25 per cent.; Behm, Hofmeyer and Wydel, 30-40 per cent.; Dorland, 30 per cent.; Parvin, 36 per cent.; Winkel, 49 per cent. But what is astonishing and to me incomprehensible, is the report of Murphy, who claims to have had 61 cases with only two deaths, and one of the two was moribund when first seen. His method is to bring on labor at the seventh month or before. I predict that Murphy will maintain during this and the next generation the honor of an isolated and lonely example of extraordinary success in the management of placenta previa.

Reynolds in his work says: "The successful termination of a case of central insertion is a feat of which any obstetrician may well be proud." According to this opinion Murphy deserves to be canonised as a benefactor of mankind.

It is doubtlessly a fact that improvements have been made in the management of placenta previa in recent times and that the death-rate has been lowered, but I cannot be made to believe that the rate of deaths will be lowered to a minimum or to a few per cent. for a long time to come, for placenta previa presents in many cases a dangerous state of things, and no matter what management may be adopted fatal results cannot altogether be prevented.

My experience in the management of placenta previa was not such as to enable me to contribute anything of importance which may be considered an improvement in its treatment. I would suggest, however, that the pregnant woman with indubitable indications of the insertion of the placenta over the os, the diagnosis of which accident can, with few exceptions, be made several months before the arrival of the time of confinement, be placed in a hospital, the interne to be instructed in the preliminaries of its management, and to send at once for the physician engaged in the case. Such arrangement should, in my opinion, supercede the induction of premature labor, a procedure the responsibility of which I should not like to shoulder. Consider a case and its possible result of induced premature labor in a case of placenta previa which may and sometimes does happen! A woman, enjoying at the time perfect health and vigor of life, and attending to her household duties as usual, but by your interference, although for a laudable purpose of saving her life, you find her a corpse the next day! Who caused her death? Comfort cannot be had in the contemplation that she would have died just the same whether interfered with before the full term of gestation or not until labor had commenced, but such consolation is very problematic and not at all certain. That the former somewhat crude method of turning and quick delivery has of late been superceded by the more gentle and safer method of Braxton Hicks, and thereby the danger in some degree lessened, but by no means overcome, I admit is true. I had but one case in which premature labor was induced, which I will mention a little further on, and that one case impressed me much with the awful responsibility the obstetrician assumes when he resorts to this method of treatment; yet, today it is practised by a number of men. Winkel, of Munich, and others are adverse to its adoption.

In post partum hemorrhage, which frequently takes place, I have confidence in swabbing the locality where the placenta had been attached, with a solution of persulphate of iron as one of the best hemostatics we possess.

I am now tempted to relate a lively scene which took place at a meeting of the Buffalo Medical Association in 1868, a sort of a picnic. Dr. Gay and myself (although I was not present at the meeting,) furnished the substantials for the entertainment. The occasion being opportune, for Dr. Gay was not the favorite with them, or rather the majority of them, and I was remembered as having some years before belonged to an opposite faction from a number of those present at this meeting ; it was, therefore, a feast for some of them to indulge in a little retaliation and they enjoyed it hugely.

Dr. Gay reported me, without my knowledge or consent, as having treated six cases of placenta previa within eight months, and that I had the full complement of fatalities that the statistics in such cases would allow, he having been my consultant and assistant in several cases and at such times doubtless learned of me some facts of the cases he reported.

Thereupon I was made the butt of unsparing criticism by most of the members. For a general practitioner to have treated six cases of placenta previa in eight months was something unprecedented and incomprehensible to them, and that I must have made a mistake in the diagnosis, although the cases of death reported they readily credited me with without question. Dr. Miner, true to his vocation, seemed to prefer a butcher knife to a scalpel in dissecting me.

Dr. Gay's turn was also on, since unguardedly he had related two cases and the management of them, but that in future he would trust longer to nature to complete labor. This was enough for Dr. White, who overhauled him severely because he did not follow the prevailing method of that time, which was to turn and deliver at once. Dr. Ring then came to the rescue with a wet blanket by stating that he had had only one case during his practice, to which he called Dr. White as assistant, but that both mother and child died. This dampened somewhat the exuberance of the placenta previa lore. They were very wise on this subject, and with a very moderate experience they knew better how to treat such cases than the accused.

It is rather late now for me to endeavor to vindicate myself against the insinuations so long ago made, but that I treated the number of cases reported and within the time mentioned and that I was able then as now to make a correct diagnosis of the cases would be a waste of words to prove.

Placenta previa is not timid ; on the contrary, it is very bold ; it does not conceal its color when you meet it, but makes its presence dangerously evident, and the doctor who fails to diagnosticate it must be lamentably defective in comprehension.

I may have made mistakes, but unless the treatment then in vogue was a mistake, I do not remember of having made any very serious one; but in that I am not alone, for, show me the physician of some practice who has never made a mistake! The most eminent make mistakes and repeat them. I witnessed once the world-renowned surgeon, Hamilton, perform the operation for stone in the bladder when no stone was to be found. This was certainly a mistake.

I have already indicated that I would make mention of a case of placenta previa in which premature labor was instituted. I reported the case in the BUFFALO MEDICAL JOURNAL, but I omitted purposely to state some facts which, when related, may have the effect of dampening somewhat the ardor which might prevail upon some physicians to adopt the method.

August 25, 1878. The woman before commencing the induction of labor was apparently in perfect health and vigor of life, attending as usual to her accustomed household duties. At 8 o'clock P. M. a catheter was inserted to bring on labor, which was followed immediately by a copious hemorrhage requiring the tampon and T bandage. I stayed in the house all night, but with the exception of using the catheter once to draw off her urine during the night there was no occasion to do anything. At 9 o'clock the next morning the first pain indicating that labor was beginning, with a sudden loss of blood, took place, and there being no certainty as to the time when active interference would become necessary, and Dr. White being out of town, I sent for Dr. Rochester, with whom he had already an understanding.

At 12 o'clock, noon, turning and delivery at once were performed by Dr. White. Ergot per mouth and hypodermically was used to arrest the post partum hemorrhage and a tampon soaked in cider vinegar was introduced far up in the vagina. All three of us had a lively spell for a time, since the woman was very weak from the shock of so sudden emptying of the uterus and in addition from the loss of blood, which was yet oozing out, notwithstanding the tampon. She rallied, however, after a while and things began to look promising. This condition lasted about three-quarters of an hour and I was left alone with the patient, during which time, however, napkin after napkin was removed considerably saturated with blood; this began to tell upon the patient and I had to resort to stimulants, raised the foot end of the bed considerably, and swabbed the part where the placenta had been attached with a solution of persulphate of iron instead of cider vinegar, which controlled the hemorrhage completely. At the same time I sent for Dr. White, who soon after arrived, took a chair a little away from the woman and observed her. By this time our patient was in a desperate condition, her pulse at the wrist was hardly perceptible and her life was ebbing, as it were.

Those of you who knew Dr. White's characteristics, his bearing, his dominant and commanding attitude, will be surprised at the statement of my observation, when he sat for a long time, seemingly unable to suggest anything, mute, despondent, the picture of anxiety.

From time to time I would apply my fingers to the radial artery of the patient with no prospect of returning improvement of the circulation. At last, however, a faint glimmer of hope began to assert itself, slowly and gradually, little by little, the artery began to fill. We commenced to feel greatly relieved when the assurance gradually began to dawn that she would recover. And she did recover, although having had already one foot in the grave. This case, more than anything else, caused an aversion in me against the induction of premature labor in placenta previa, and I doubt if Dr. White ever after the experience of this case advocated its adoption. I have the vanity to believe that Dr. White on this occasion imbibed the idea that I knew something of placenta previa, for after this he was my friend, and in a meeting of the Medical Society of the County of Erie, in 1881, he canvassed the members for my presidency without my solicitation.

I have one more case of placenta previa to relate briefly and then finish. It is one of my last cases.

Some years ago I was called by a physician to a case and on my arrival I found the woman had already lost considerable blood. On examination I detected the placenta partially inserted over the os and by pushing it a little aside by my finger, I could feel a little farther up the head of the fetus. I considered the case a proper one for forceps delivery, and without loss of time succeeded in applying the forceps and delivered the child. The woman made a good recovery.

The entire subject of placenta previa is yet a tangle and a conflict of opinions as regards its management and treatment and the course for which we think we have the best of reason to pursue today, is subject to be condemned tomorrow. The obstetrician in no other cases of midwifery is burdened with so much responsibility or is required to exercise more sound judgment than in these cases, since every case has its peculiarities which must be met, and in spite of all our knowledge, reason, earnest endeavor and carefulness, we are frequently doomed to disappointment.

309 ELMWOOD AVENUE.

